# IMicAP: an intelligent microbial application platform for knowledge-driven microbiome research

**DOI:** 10.3389/fmicb.2026.1955928

**Published:** 2026-10-01

**Authors:** Chaoyu Zhu, Xing Wang, Lihui Feng, Mingzhang Xu, Zhengkun Huang, Weijie Chen, Lei Liu

**Affiliations:** 1 Intelligence Medicine Institute, Fudan University, Shanghai, China; 2 Shanghai Lishan Biotech Co., Ltd., Shanghai, China; 3 Center for Gut Microbiome, Metabolism and Immunity, Shanxi Bethune Hospital, Shanxi Academy of Medical Sciences, Tongji Shanxi Hospital, Third Hospital of Shanxi Medical University, Taiyuan, China; 4 Avon Old Farms School, Avon, CT, United States

**Keywords:** artificial intelligence, data integration, host–microbe interactions, knowledge graph, microbiome research

## Abstract

**Background:**

Microorganisms play a pivotal role in health and disease, yet microbial research is hindered by fragmented data, heterogeneous knowledge representation, and a lack of integrated analytical tools. Existing databases and knowledge graphs often focus narrowly on genomic information, leaving critical metabolomic and literature-based associations underdeveloped.

**Results:**

We developed the Intelligent Microbial Application Platform (IMicAP), an AI-driven, open-access web platform that integrates 12 microbial databases and over 35 million articles. IMicAP delivers 5 core modules: (1) Knowledge Query module for rapid data retrieval; (2) Knowledge Graph module for multi-hop reasoning and hypothesis generation; (3) Intelligent Question-Answering module for professional natural language dialogue in the field of microbiology; (4) Genome Browser module for genomic visualization; and (5) 16S rRNA Sequencing Analysis module for zero-code microbial bioinformatics analysis. Using curated microbe-disease and microbe-small molecule relations extracted with biomedical language models, IMicAP enables context-aware exploration of microbial relations.

**Conclusion:**

IMicAP (available at: https://www.imicap.com:8443) is the first comprehensive, open-access, AI-powered microbial platform designed to bridge the gap between fragmented microbial data and actionable biological insights. It directly addresses 4 key limitations in existing resources: (1) Fragmented analysis without integrated workflows; (2) Narrow disease scope covering only one or two disease types; (3) Incomplete knowledge from restricted data sources; and (4) neglect of metabolomic information. IMicAP serves as a versatile resource for advancing microbiome research and translational discoveries. Future development focus on expanding microbial coverage, enhancing reasoning scalability, and evolving toward a dual knowledge-and-data-driven architecture to further support mechanistic discovery in microbial systems biology.

## 1 Introduction

Microbes are essential for maintaining both ecological balance and human health (Banerjee and Van Der Heijden, 2023; Lynch and Pedersen, 2016). The human gut microbiota, a complex ecosystem comprising trillions of microorganisms, plays central roles in digestion, immune regulation, metabolism, neurological function, and disease defense (Arumugam et al., 2011; Zhou et al., 2024). It supports nutrient extraction, vitamin synthesis, immune homeostasis, and intestinal barrier integrity (Valdes et al., 2018; Di Tommaso et al., 2021). Disruption of this symbiosis is linked not only to gastrointestinal disorders but also to systemic conditions through the gut-X axis, affecting multiple organs including the brain, cardiovascular system, liver, kidneys, lungs, skin, and skeletal system (Lin et al., 2025). However, research in this area faces substantial challenges: the gut microbiota generates multi-layered omics datasets from hundreds to thousands of microbial species, posing significant computational and analytical demands (Zhernakova et al., 2016; Shi et al., 2022). Furthermore, intricate bidirectional host-microbiota communication, and multi-directional microbe-microbe interactions, shaped by diverse factors and physiological states, complicates the identification of reliable health-related biomarkers (Gilbert et al., 2018; Zhang F. et al., 2022).

In response to these challenges, big data and Artificial Intelligence (AI) methods have become increasingly important for mining large-scale microbiota data, enabling refined health assessments and advancing precision microbial therapies (Knight et al., 2018; Lindell et al., 2022). Nevertheless, microbiological data resources remain fragmented due to the sheer volume of data, diversity of data modalities, and lack of standardization. These limitations impede effective data integration, which is essential for realizing the full scientific and translational potential of microbiota research (Quince et al., 2017; Lagier et al., 2018; Mitchell et al., 2020).

Currently, a wide array of comprehensive microbial databases, such as the Human Microbiome Project (HMP) (Turnbaugh et al., 2007) and GMRepo (Dai et al., 2022), provide extensive collections of microbial sequencing data but often lacking systematically organized knowledge about the microorganisms themselves. Meanwhile, established microbial knowledge bases (e.g., NCBI Taxonomy (Schoch et al., 2020), BioCyc (Karp et al., 2019), MetaCyc (Caspi et al., 2019), and EcoCyc (Karp et al., 2025)) and knowledge graphs (e.g., FGMDI (Zhang et al., 2024), MMiKG (Sun et al., 2023), KetPath (Liu T. et al., 2024), and KGVHI (Pan et al., 2024)) have been constructed under diverse architectures and standards. This heterogeneity creates a critical analytical bottleneck: the inability to integrate, query, and analyze across different knowledge sources and data modalities in a unified manner. This bottleneck manifests as 4 specific limitations:

(1) Fragmented analysis: Reliance on conventional search functions without integrated analytical workflows or intelligent query capabilities limits exploratory analysis;
(2) Narrow disease scope: Most resources focus on only one or two disease types (e.g., mental disorders), severely limiting generalizability.
(3) Incomplete knowledge: Frequent reliance on restricted data sources, such as limited databases or sparse literature, leads to incomplete knowledge representation and less convincing conclusions.
(4) Neglect of metabolomic information: These resources focus primarily on microbial genomic knowledge while overlooking metabolomic information (Thompson et al., 2021). Yet microbial metabolites are central to host–microbe interactions, with their complex structures and functions playing critical roles in host physiology and disease (Rooks and Garrett, 2016). However, deciphering these relations remains challenging due to the high dimensionality and inherent variability of metabolite data (Bartmanski et al., 2023).

To address these constraints, we introduce the Intelligent Microbial Application Platform (IMicAP), a knowledge-driven platform that does not aim to replace existing specialized databases. Instead, its novelty lies in two specific aspects: first, extracting previously underutilized knowledge from the scientific literature, and second, building an integrated platform that synthesizes heterogeneous data sources. By leveraging structured knowledge from established databases while further uncovering often-overlooked, unstructured associations among microbes, diseases, and small molecules from the published literature, IMicAP dissolves data silos and accelerates research into microbial-host interactions. The platform is implemented as an openly accessible web service at: https://www.imicap.com:8443 and provides 5 core functional modules for microbiological research: (1) Knowledge Query, (2) Knowledge Graph, (3) Intelligent Question & Answering (Q&A), (4) Genome Browser, and (5) 16S rRNA Sequencing Analysis. These modules collectively enable researchers to systematically explore metabolite-centric relationships, formulate mechanistic hypotheses, and identify potential microbial or metabolic biomarkers across various diseases. By establishing a transformative framework, IMicAP significantly improves the efficiency of knowledge retrieval, analysis, and learning in microbiology, facilitates the elucidation of key pathways within complex microbial systems, and advances translational insights into both health and disease.

## 2 Results

### 2.1 IMicAP overview

We systematically selected 12 high-quality databases from a global collection of 6,047 microbial-related databases, applying core selection criteria including knowledge relevance, data accessibility, update frequency, and authoritative standing. These encompass NCBI-Taxonomy (Schoch et al., 2020), BioCyc (Karp et al., 2019), MetaCyc (Caspi et al., 2019), EcoCyc (Karp et al., 2025), UniProt (UniProt Consortium, 2019), Gene Ontology (GO) (Gene Ontology Consortium, 2015), BV-BRC (Olson et al., 2023), EMBL-EBI (Madeira et al., 2022), BacDive (Schober et al., 2025), hGMB (Liu et al., 2021), OMIM (Amberger and Hamosh, 2017), and MicrobeWiki (Josowitz, n.d.). Additionally, it integrates extensive biomedical literature data from PubTator3 (Wei et al., 2024), which contains annotations for over 35 million PubMed abstracts and PMC full-text articles. Following this, multi-dimensional data undergo a comprehensive pipeline comprising downloading, cleaning, standardization, integration, and harmonization. The IMicAP platform ultimately delivers 5 core functional modules for microbiological research: Knowledge Query, Knowledge Graph, Intelligent Q&A, Genome Browser, and 16S rRNA Sequencing Analysis (Figure 1). Detailed operation guidelines are provided in Supplementary Results S1.

**Figure 1:**
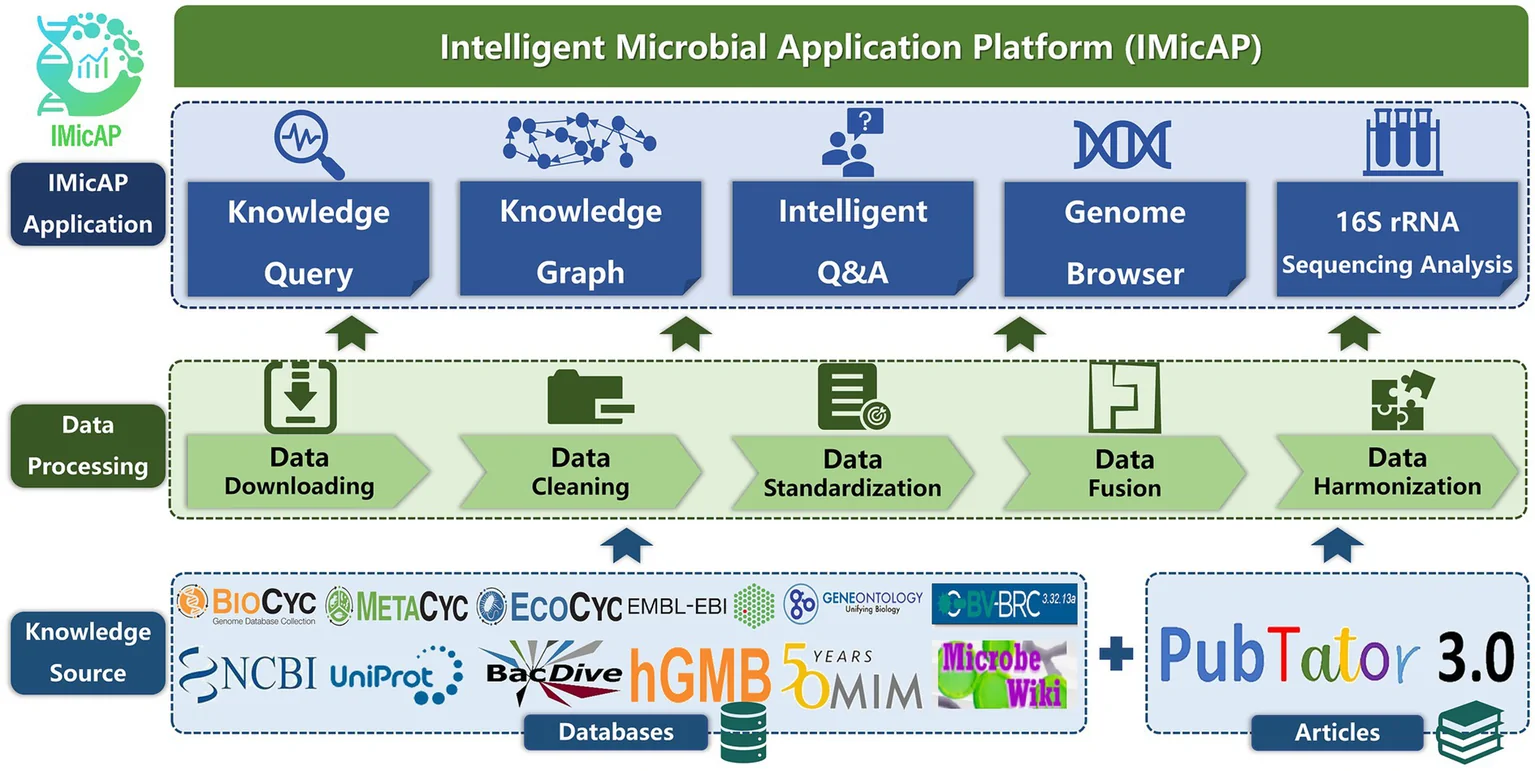
The workflow of IMicAP. Including knowledge sources, data processing steps, and applications.

### 2.2 Data summary

IMicAP includes a total of 594 microbe species (Supplementary Table S1), spanning 6 kingdoms, 19 phyla, 26 classes, 42 orders, 81 families, and 227 genera (Figure 2A). Among them, 109 species correspond to baseline microbial taxa from the healthy human gut (King et al., 2019), while the remaining 485 species are sourced from hGMB (Liu et al., 2021) and are widely distributed in the Chinese population. It should be noted that these microbial species are not restricted to gut commensals, but also encompass taxa originating from the oral cavity, diet, water, and other environmental exposures, reflecting the natural composition of fecal samples. All microbial names were standardized according to the List of Prokaryotic names with Standing in Nomenclature (LPSN) (Parte, 2014), with historical synonyms mapped to their current scientific names.

**Figure 2:**
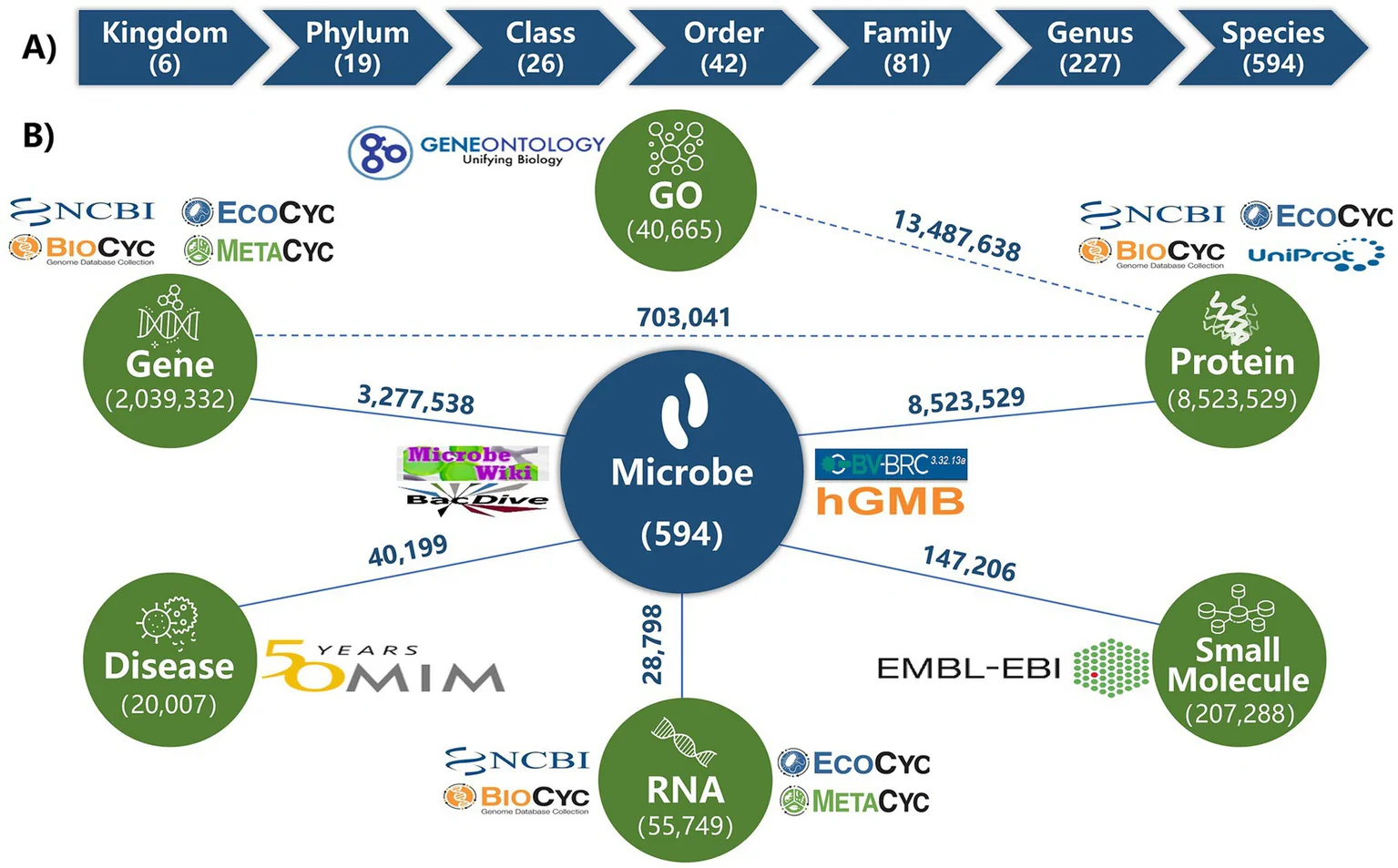
Data summary of IMicAP. **(A)** Taxonomic hierarchy statistics diagram. **(B)** Knowledge architecture, sources, and statistics. Entity nodes (e.g., proteins) are defined within each organism’s context and are not deduplicated across microbes.

By integrating multi-modal data, the platform establishes a sophisticated association network that enables comprehensive analysis of complex biological systems. Structured around 594 microbial species, IMicAP encompasses 7 entity types and 7 relation types (Figure 2B). All knowledge is maintained for timeliness and relevance through regular updates of databases and literature, and is displayed in real-time via the “Data Summary” section on the IMicAP website. This section also provides extensive statistics on Proteins, RNA, Morphology, Cultivation, Metabolism, Isolation Sources, Molecular Biology & Pathogenicity, and Tests & Profiles (Supplementary Table S2).

### 2.3 Knowledge Query

The Knowledge Query module within IMicAP simplifies access to complex, multi-source, and heterogeneous microbial data by enabling researchers to perform one-click, keyword-based searches that instantly retrieve and integrate diverse data types from multiple sources. For example, a search for *Escherichia coli* returns a comprehensive profile systematically organized into dedicated sections such as “General Information,” “Morphology,” “Culture and Growth Conditions,” “Physiology and Metabolism,” “Isolation, Sampling and Environment,” “Sequence,” “Biotechnological Applications,” “Ecology and Pathogenesis,” “Clinical Aspects,” “Safety,” “Experimental Data,” “Current Research,” and “References” (Figure 3A). By seamlessly integrating these multi-scale data layers, the Knowledge Query module offers a unified platform for microbial data exploration, significantly accelerating data retrieval and hypothesis generation while upholding high standards of data accuracy and timeliness. Retrieval items for other entity types are detailed in Supplementary Table S3.

**Figure 3:**
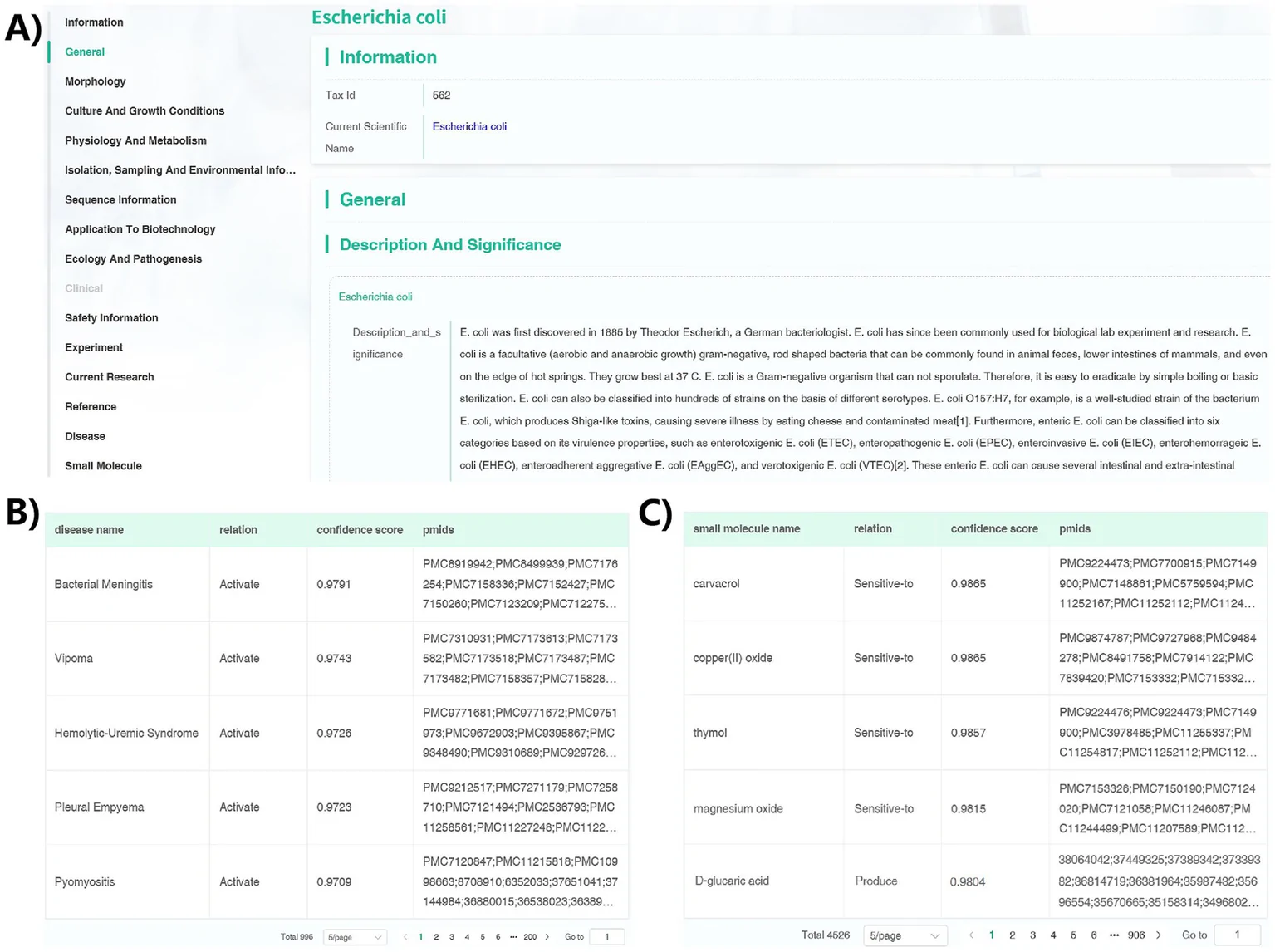
The Knowledge Query module within IMicAP. **(A)** A search for *Escherichia coli* returns a comprehensive profile systematically organized into dedicated sections. **(B)** Microbe-associated diseases along with their relations and confidence scores. **(C)** Microbe-associated small molecules along with their relations and confidence scores.

Furthermore, the associations between these microbes and relevant diseases (relation as “Activate” or “Inhibit”) and small molecules (relation as “Produce,” “Consume,” “Sensitive-to,” or “Resistant-to”) were systematically extracted from PubTator3 articles (Wei et al., 2024). This effort addresses a significant void in current microbial knowledge bases, which have traditionally lacked these detailed, interaction-focused annotations. This endeavor effectively fills a gap in current microbial knowledge bases, particularly regarding microbial metabolomic information. IMicAP evaluated each association by integrating multiple evidence factors, including the number of supporting articles, publication year, journal impact factor, and paragraph relevance scores, to compute a quantitative confidence score. This process extends the conventional triple-based representation to a quadruple format. The final associations are ranked in descending order of confidence, with all supporting PubMed Identifiers (PMIDs) provided for reference (Figures 3B,C). The complete extracted quadruples are provided in Supplementary Table S4.

### 2.4 Knowledge Graph

To meet the critical need for comprehensive analysis of host-microbiota interactions, the Knowledge Graph module within IMicAP integrates heterogeneous biological data into a unified network framework. This framework enables efficient relation queries and supports multi-hop relation analysis, such as investigating third-order correlations (e.g., Gene-Microbe-Disease and GO term-Protein-Microbe). Researchers can thereby visually explore complex biological network relationships. The system facilitates traversal across various node types, with edges representing diverse relations between nodes. There are 3 core query methods: (1) Adjacent search quickly finds all associated entities directly connected to the target node; (2) Keyword-to-Category search explores the hierarchical relation between the input entity and all nodes in a given category; and (3) Keyword-to-Keyword search reveals all potential paths between any two target nodes. Each query method adopts a visual interactive design, supporting users to explore knowledge associations interactively.

To demonstrate, the Knowledge Graph module in IMicAP reveals that *Enterobacter asburiae* synthesizes several bioactive small molecules, including indole-3-acetic acid, (R)-lactic acid, L-tryptophan, and butyric acid (Figure 4A). Notably, other microbes known to inhibit Inflammatory Bowel Disease (IBD), such as *Lactobacillus* [e.g., *Lactobacillus gasseri* (Zhang W. Q. et al., 2022), *Lactobacillus johnsonii* (Zhou et al., 2025), and *Lactobacillus helveticus* (Cui et al., 2025)] and *Bifidobacterium* [e.g., *Bifidobacterium bifidum* (Liu et al., 2023), *Bifidobacterium longum* (Yao et al., 2021), and *Bifidobacterium dentium* (Zhao et al., 2021)] are also reported to produce these same 4 small molecules. Given this shared biosynthetic profile identified by the Knowledge Graph, we hypothesized that *Enterobacter asburiae* may similarly exhibit IBD-inhibitory activity. Indeed, subsequent literature has confirmed this conjecture through murine model experiments (De et al., 2021). This example illustrates how our Knowledge Graph module can surface under-explored associations and translate them into experimentally testable hypotheses for downstream validation.

**Figure 4:**
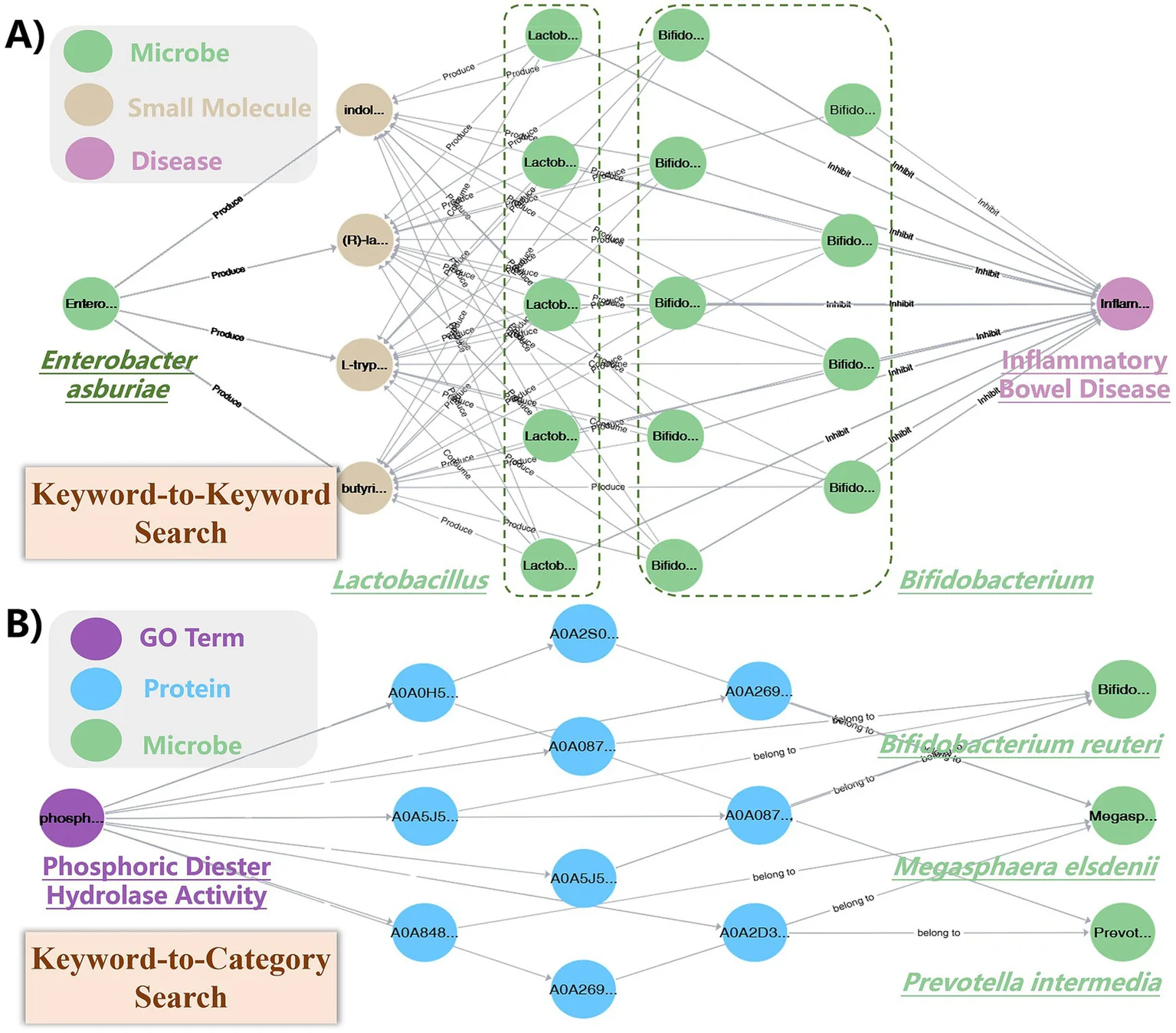
The Knowledge Graph module within IMicAP. **(A)** Microbe (*Enterobacter asburiae*)-Small Molecule-Microbe-Disease (IBD) association analysis by Keyword-to-Keyword search. **(B)** GO term (phosphoric diester hydrolase activity)-Protein-Microbe (*Bifidobacterium reuteri*, *Megasphaera elsdenii*, and *Prevotella intermedia*) association analysis by Keyword-to-Category search.

The Knowledge Graph module in IMicAP further demonstrates its utility by enabling queries for microbes associated with specific GO terms. The GO term serves as a standardized resource for annotating gene products across species, categorizing them into molecular function, biological process, and cellular component (Gene Ontology Consortium, 2015; Reijnders and Waterhouse, 2021). Leveraging this, the system rapidly identifies functionally similar microbes, such as those involved in phosphoric diester hydrolase activity, and facilitates the analysis of their biological linkages, as exemplified by a query that identified three species of functional interest: *Bifidobacterium reuteri*, *Megasphaera elsdenii*, and *Prevotella intermedia* (Figure 4B). Finally, the interactive interface allows for in-depth microbial profiling through selective examination and Knowledge Query operations, thereby enabling thorough functional characterization and comparative studies.

### 2.5 Intelligent Q&A

Conventional knowledge retrieval systems are constrained by their reliance on rigid and fragmented query structures, which can hinder data accessibility and research efficiency. To address these challenges in microbiota research, the Intelligent Q&A module within IMicAP bridges complex biological datasets and researcher needs by integrating: (1) Qwen3-Max-Thinking-Preview’s natural language generation capabilities (Yang et al., 2025); (2) A Retrieval-Augmented Generation (RAG) architecture for information retrieval (Amugongo et al., 2025); (3) Our curated microbial knowledge base. This synergistic combination facilitates context-aware dialogue between researchers and our intelligent agent system, which supports improved efficiency in microbial knowledge acquisition and hypothesis generation, while helping to ensure that responses remain grounded in domain-specific expertise. This makes the approach particularly useful for knowledge-intensive question answering in microbiology where consistent and well-founded responses are important.

Taking the simple query “Introduce about *Escherichia coli*.” and “Which microorganisms are more likely to cause infection?” as illustrative examples, it is clear that the Intelligent Q&A module enhanced with RAG from a specialized microbial knowledge base produces more comprehensive, logically organized, and contextually enriched responses compared to those generated by Qwen3-Max-Thinking-Preview alone (Supplementary Result S2). By leveraging authoritative and current external knowledge, the system provides a precise, well-structured, and insightful overview that effectively addresses the limitations of conventional retrieval systems, which are fundamentally constrained by rigid and fragmented query structures that substantially hinder both data accessibility and research efficiency.

### 2.6 Genome Browser

To facilitate the comprehensive exploration of microbial genome architectures, the Genome Browser module within IMicAP is specifically designed for genomic data visualization, annotation, and interpretation (Nassar et al., 2023). This sophisticated tool provides an intuitive visualization of microbial genome sequences along with their annotation features, enabling researchers to clearly examine critical genomic elements such as gene structures, coding sequences, and regulatory regions (Figure 5). Integrating diverse data types with dynamic scaling functionality, the Genome Browser module allows researchers to efficiently navigate to specific genomic regions by searching for gene names or chromosomal coordinates. Featuring smooth zooming capabilities, it also supports seamless exploration across multiple scales, from single nucleotides to entire chromosomes.

**Figure 5:**
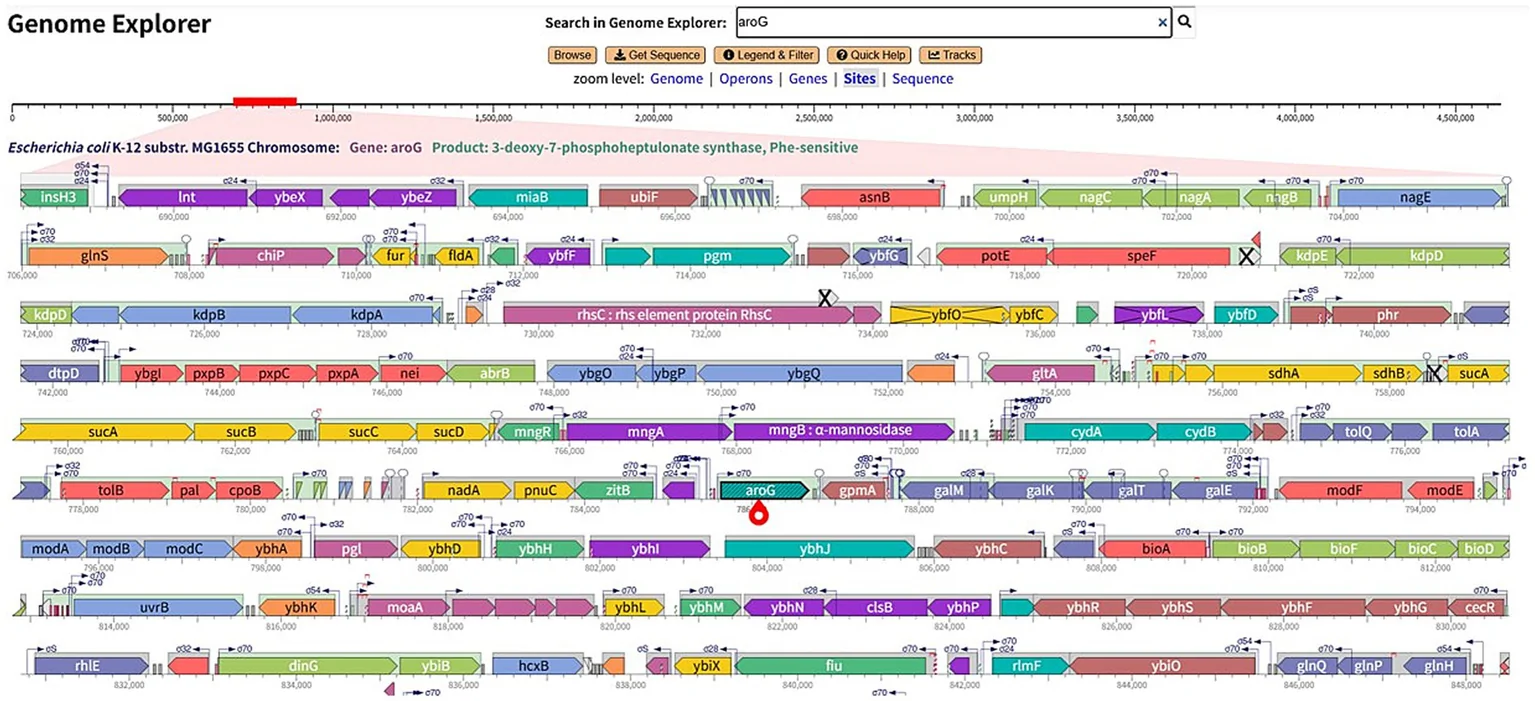
The Genome Browser module within IMicAP. Taking the *aroG* gene as an example, its location within the *Escherichia coli* K-12 genome is indicated by a red arrow (Lin et al., 2014).

### 2.7 16S rRNA sequencing analysis

16S rRNA sequencing has become the most widely adopted method for microbial community characterization in both academic and industrial fields (Church et al., 2020). However, the subsequent bioinformatic analysis presents a significant hurdle. Traditional workflows demand specialized computational expertise that many researchers lack. Outsourcing to sequencing service providers often restricts flexibility, as they typically offer standardized, template-based reports that do not support customized, hypothesis-driven exploration aligned with specific research objectives.

To address these challenges, the 16S rRNA Sequencing Analysis module within IMicAP provides a streamlined, zero-code workflow that converts raw sequencing data into comprehensive omics-level insights. To protect patient privacy, users uploading compressed FASTQ data should label each sample with a unique code and avoid including any clinical information in the files. The platform integrates 8 dedicated modules and 25 analytical components to support multidimensional data exploration (Figure 6A). It enables key microbiome analyses including community structure profiling, phylogenetic inference, biomarker identification, and microbe-environment correlation studies, with fully customizable parameters to accommodate diverse research needs while maintaining robust analytical capabilities (Regueira-Iglesias et al., 2023).

**Figure 6:**
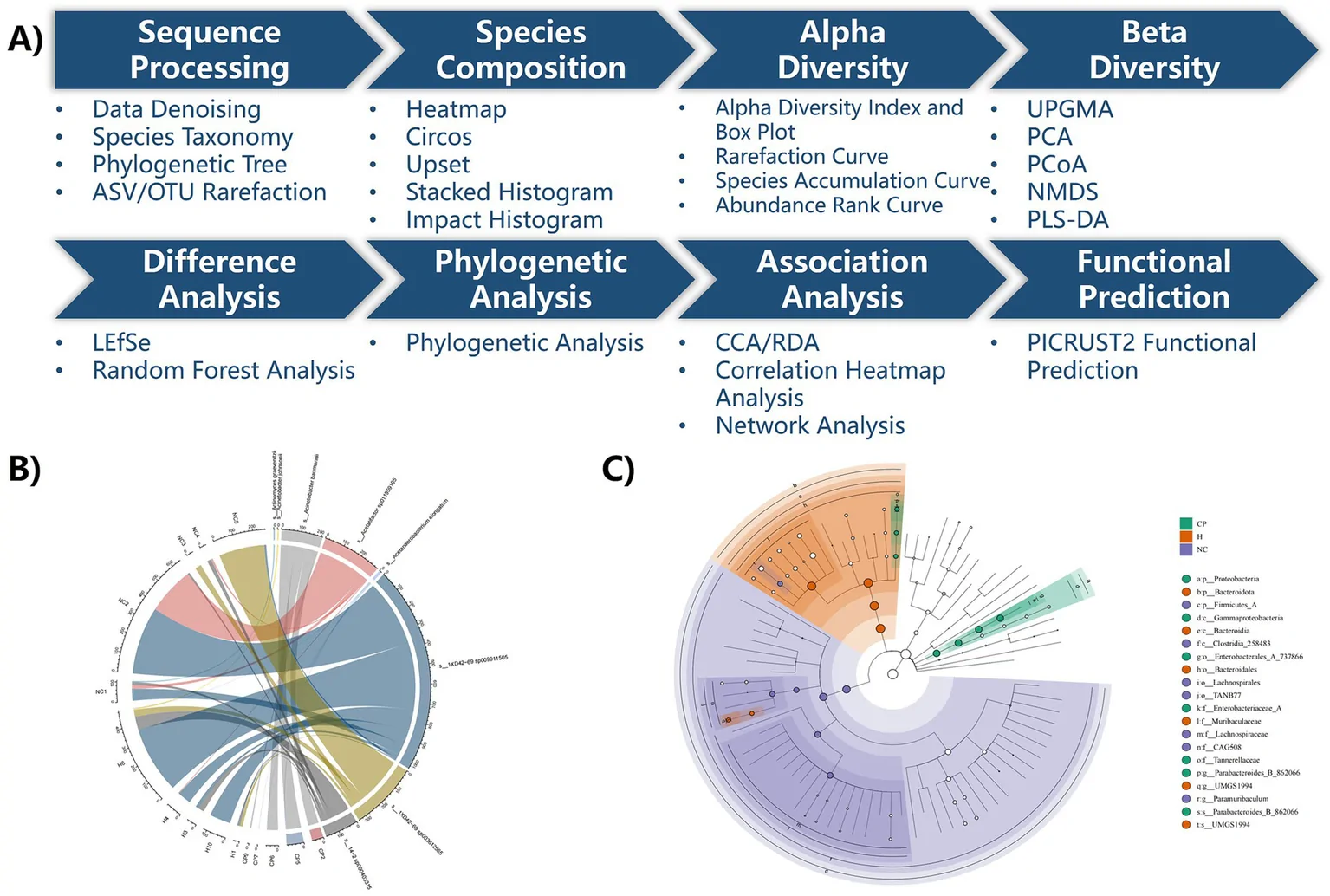
The 16S rRNA Sequencing Analysis module within IMicAP. **(A)** 8 dedicated analysis modules and 25 analytical components. **(B)** Circos diagram visualizing Chinese population data from GMRepo (Dai et al., 2022). The left semicircle displays genus-level relative abundance of microbial species across samples, while the right semicircle illustrates the proportional distribution of each genus among different samples. **(C)** LEfSe cladogram depicting Chinese population data from GMRepo (Dai et al., 2022), illustrating evolutionary relations among microbe groups. Each node represents a common ancestor, while branches denote evolutionary divergence paths.

Understanding microbial community structure is critical in many applications and can be evaluated through Species Composition analysis. A Circos (Krzywinski et al., 2009) diagram effectively visualizes dynamic changes, species counts, individual abundances, dominance, rarity, and other compositional features (Figure 6B); To compare community composition or abundance across sample groups and identify statistically significant differences, Differential Analysis can be applied. This approach detects distinct microbial patterns between groups without relying on identical sampling conditions, while preserving the integrity of original compositions by LEfSe (Chang et al., 2022) cladogram (Figure 6C).

## 3 Materials and methods

### 3.1 Database knowledge curation and harmonization

The database knowledge of IMicAP was constructed through systematic integration of 12 authoritative microbial databases, including NCBI-Taxonomy, BioCyc, MetaCyc, EcoCyc, UniProt, GO term, BV-BRC, EMBL-EBI, BacDive, hGMB, OMIM, and MicrobeWiki. These resources were obtained via standard FTP or batch downloads to ensure broad genomic and functional coverage. Through rigorous curation, we resolved issues like hierarchical data structures, strain synonym redundancies, and inconsistencies, while retaining data relevant to 594 target gut microbiota species.

All data underwent extensive processing pipelines before being stored in a distributed data lake architecture (Singh and Ahmad, 2019). This multi-layered integration forms the core framework of the IMicAP Knowledge Base, supporting robust data representation and accessible querying. Subsequent standardization aligned key data fields such as microbial nomenclature, taxonomic hierarchies, and gene identification systems with international bioinformatics standards and established research practices. The resulting knowledge base provides a meticulously curated and standardized resource tailored for advanced microbiome research applications.

### 3.2 Literature knowledge extraction

The literature knowledge base for IMicAP was systematically constructed by retrieving and decompressing pre-processed raw XML files from the PubTator3 dataset available on the NCBI FTP site (Wei et al., 2024). The initial corpus consisted of 38,274,953 annotated PubMed abstracts and full-text articles from PMC. From this corpus, we identified 1,279,960 articles containing at least one microbial entity, as defined by NCBI Taxonomy IDs, for subsequent annotation and relation extraction.

We randomly selected 2,600 qualifying paragraphs for each entity pair type (microbe-disease and microbe-small molecule) for relation annotation, where microbe-disease relations were labeled as “Activate,” “Inhibit,” or “Unrelated,” while microbe-small molecule relations were labeled as “Produce,” “Consume,” “Sensitive-to,” “Resistant-to,” or “Unrelated.” To effectively utilize PubTator3’s existing annotations, each entity in the text was demarcated with specific boundary tokens: microbes were enclosed within “@gm$” and “@/gm$,” diseases within “@di$” and “@/di$,” and small molecules within “@sm$” and “@/sm$.” For quality control, a two-stage automated labeling pipeline was implemented using GPT-4o (Hurst et al., 2024) and DeepSeek-v3 (Liu A. et al., 2024). Following this automated process, all 2,600 paragraphs per relation type were manually reviewed to ensure consistency, and were subsequently retained for relation extraction. Of these, 2,000 paragraphs were used for training, 500 for the validation (dev) set, and 100 as the held-out test set (Supplementary Tables S5, S6).

We formulated relation annotation as a classification task and conducted experimental comparisons using 4 biomedical pre-trained language models as encoders: BioBERT (Lee et al., 2020), SciBERT (Beltagy et al., 2019), PubMedBERT (Gu et al., 2021), and BioLinkBERT (Yasunaga et al., 2022). To address label imbalance, we evaluated 4 sampling strategies during training: (1) NONE for original data distribution; (2) SUB for sub-sampling majority classes; (3) SUB-UP for combining sub-sampling of majority classes and up-sampling of minority classes; (4) UP for up-sampling minority classes exclusively.

Ultimately, the optimal configuration for each task was selected on the dev split by Macro-F1 score (Table 1; Supplementary Table S7). In the microbe–disease 3-class task, PubMedBERT + NONE attained the highest dev Macro-F1 (0.8094) and, applied to the held-out test set, a Macro-F1 of 0.7578; correspondingly, in the microbe–small molecule 5-class task, BioLinkBERT + SUB-UP attained the highest dev Macro-F1 (0.7939) and a test-set Macro-F1 of 0.7168. These two selected models were subsequently used to extract relations from all relevant paragraphs.

**Table 1:** Literature knowledge relation extraction performance.

Language model	Sampling strategy	Macro-F1 score			
		Microbe-disease		Microbe-small molecule	
		Dev	Test	Dev	Test
BioBERT	NONE	0.7582	0.7243	0.7531	0.6860
	SUB	0.7412	0.7342	0.7501	0.6587
	SUB-UP	0.7562	0.7151	0.7487	0.6551
	UP	0.7529	0.7063	0.7495	0.6289
SciBERT	NONE	0.7692	0.7377	0.7822	0.6923
	SUB	0.7609	0.7268	0.7701	0.7064
	SUB-UP	0.7647	0.7458	0.7685	0.6517
	UP	0.7710	0.7382	0.7802	0.7131
PubMedBERT	NONE	**0.8094**	**0.7578**	0.7925	0.6912
	SUB	0.8078	0.7450	0.7766	0.6899
	SUB-UP	0.8069	0.7432	0.7902	0.6790
	UP	0.8048	0.7539	0.7833	0.6897
BioLinkBERT	NONE	0.7961	0.7408	0.7835	0.7162
	SUB	0.7923	0.7498	0.7807	0.6900
	SUB-UP	0.7952	0.7106	**0.7939**	**0.7168**
	UP	0.7922	0.7429	0.7849	0.6892

The model combination highlighted with underlining and bolding denotes the best performance on the dev and test set.

### 3.3 Confidence score definition

PubTator3 knowledge is automatically extracted from articles with the inevitable extraction noise. Therefore, we calculate a confidence score for each triplet as a quadruple by source information to measure its uncertainty. Assume triplet (h,r,t) has n supporting articles, $y_{i}$ , $j_{i}$ , and $s_{i}$ denotes the publication year, the journal impact factor and the SoftMax score given by relation extraction model, respectively, for the $i_{\mathrm{th}}\;(1\leq i\leq n)$ article source. Some articles may have multiple potential paragraphs for the same entity pair, calculate their average SoftMax score instead. The confidence score for non-unrelated triplet is defined as:


$$S_{\left(h,r,t\right)}=k_{n}\frac{\sum_{i=1}^{n}k_{y_{i}}k_{j_{i}}s_{i}}{n}$$

where $k_{n}$ , $k_{y_{i}}$ , and $k_{j_{i}}$ represents the monotonically increasing weighting coefficient based on n , $y_{i}$ , and $j_{i}$ , respectively. Finally, we linearly normalize all scores to [0.6000, 0.9999] and obtain the quadruple (Figure 7).

**Figure 7:**
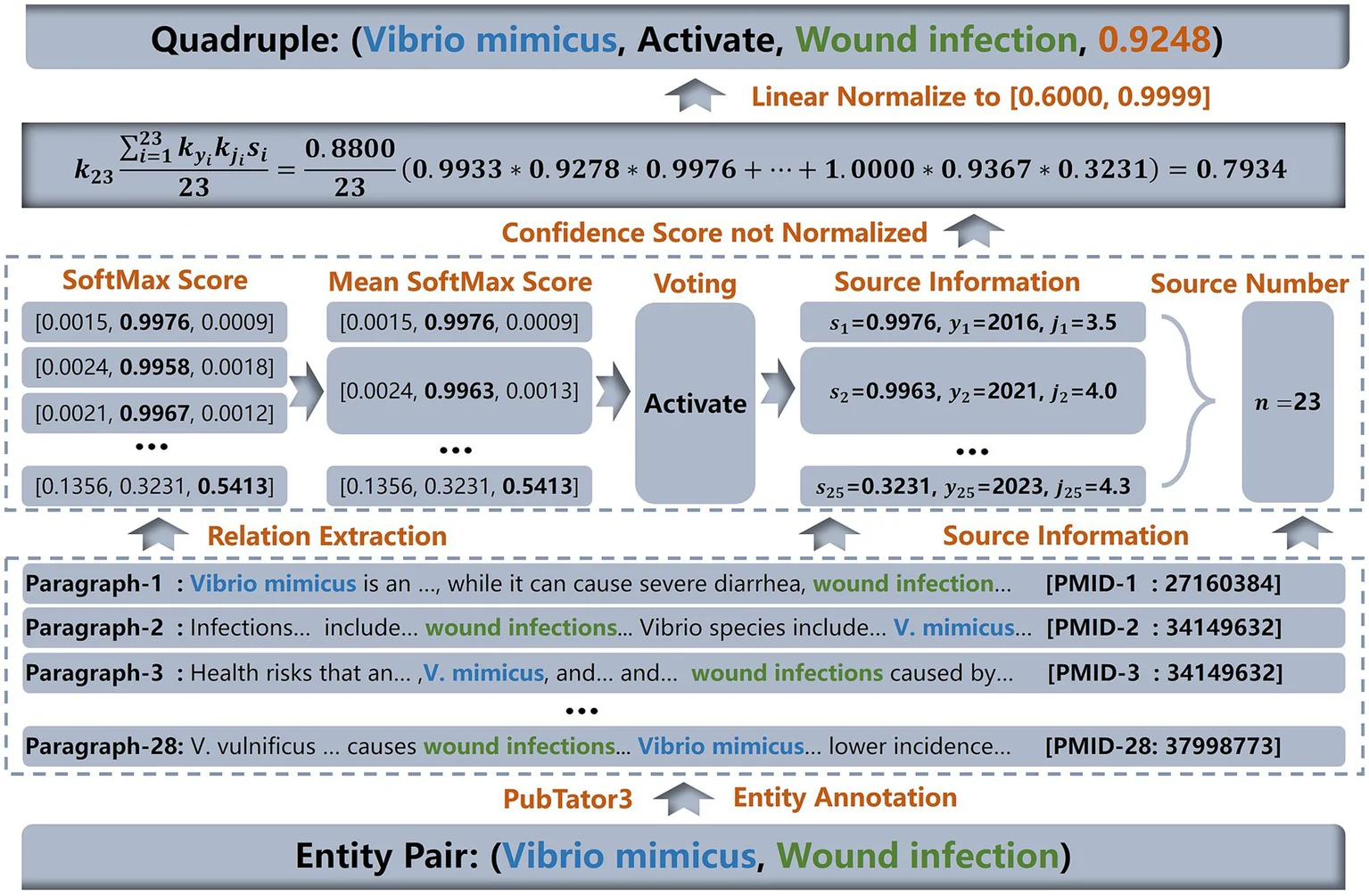
Confidence score calculation process. Taking (*vibrio mimicus*, wound infection) pair as an example, it has 23 article sources and 28 annotated potential paragraphs from PubTator3 (Wei et al., 2024). After relation extraction and confidence score calculation, we obtain the quadruple: (*vibrio mimicus*, activate, wound infection, 0.9248).

### 3.4 Development of the IMicAP database and platform

IMicAP employs a sophisticated hierarchical architecture comprising 3functionally optimized layers: 1) An interactive user interface layer; 2) A comprehensive application processing layer; and 3) A robust data management layer. The user interface layer enables flexible multi-terminal access through dedicated PC clients and an administrative console. It leverages Nginx’s advanced load-balancing capabilities and intelligent static resource allocation to maintain high system stability under high-concurrency conditions (Reese, 2008).

The data management layer adopts an innovative hybrid database architecture, where: (1) MySQL manages mission-critical structured data, including system configurations and user profiles (Ongo and Kusuma, 2018); (2) MongoDB is responsible for flexible entity storage (Ongo and Kusuma, 2018); (3) Neo4j handles intricate knowledge-graph relations (Miller, 2013); and (4) ElasticSearch maintains high-precision full-text search indices (Kathare et al., 2020). This modular design not only meets the rigorous requirements of computational biology research, but also supports diverse analytical workflows, offering a comprehensive and high-performance solution for microbial systems biology.

### 3.5 Development of the 16S rRNA sequencing analysis pipeline

For the 16S rRNA Sequencing Analysis module, we constructed and deployed an integrated bioinformatics pipeline based on QIIME 2 (Srivastava et al., 2024). In this workflow, sequence denoising constitutes a critical initial step toward accurate microbial community profiling. To this end, we incorporated the DADA2 algorithm (Callahan et al., 2016), which applies a parametric error model to systematically eliminate PCR-induced artifacts and correct sequencing errors directly from raw amplicon reads. This process yields high-resolution Amplicon Sequence Variants (ASVs) that represent biologically discrete taxa. In contrast to conventional operational taxonomic unit clustering methods, which typically rely on fixed similarity thresholds, the ASV approach achieves single-nucleotide resolution. This refinement significantly reduces the overestimation of microbial diversity and enables more precise tracking of taxonomic lineages across samples. The entire denoising process is fully automated as a one-click module, ensuring analytical reproducibility and user-friendly operation.

## 4 Discussion

In recent years, research institutions have increasingly recognized the pivotal role of microorganisms in biomedical and clinical studies, which has driven the creation of specialized microbial platforms. Yet, no current platform combines all 5 core modules of IMicAP: Knowledge Query, Knowledge Graph, Intelligent Q&A, Genome Browser, and 16S rRNA Sequencing Analysis (Table 2). The core strengths of IMicAP are reflected in several key advantages:

(1) IMicAP integrates microbial knowledge from multiple sources, serving as an open-access online platform with strong practicality that effectively enhances research efficiency for microbiologists.
(2) IMicAP leverages a dedicated microbial knowledge base to implement a RAG-enhanced Qwen3 model, delivering accurate, domain-specific Q&A capabilities in microbiology.
(3) IMicAP provides zero-code Gene Browser and 16S rRNA Sequencing Analysis tools without requiring the upload of sample clinical information, thereby eliminating data security concerns and enabling medical students and clinicians to perform flexible, non-templated independent analyses.
(4) IMicAP incorporates a wealth of distinctive microbial metabolomic knowledge, addressing a key gap in existing platforms while providing one of the most highly valued resources for microbiology researchers.

**Table 2:** Advantages and disadvantages of IMicAP compared with similar platforms.

Similar platforms		Compare with other platforms	
Category	Platform name	IMicAP’s advantages	IMicAP’s disadvantages
Comprehensive Microbe Platform	None	-	-
Microbe Database	HMP (Turnbaugh et al., 2007)	Rich microbe knowledge Explainable microbe knowledge reasoning Integrated microbial analysis	Lack of clinical sequencing data
	GMRepo (Dai et al., 2022)		
Biomedicine Knowledge Base	NCBI-Taxonomy (Schoch et al., 2020)	Focused microbial knowledge Cross-platform knowledge system Integrated microbial analysis	Limited extensibility of knowledge
	BioCyc (Karp et al., 2019)		
	MetaCyc (Caspi et al., 2019)		
	UniProt (UniProt Consortium, 2019)		
	Gene Ontology (Gene Ontology Consortium, 2015)		
	EMBL-EBI (Madeira et al., 2022)		
	OMIM (Amberger and Hamosh, 2017)		
Microbe Knowledge Base	EcoCyc (Karp et al., 2025)	Standardized knowledge structure Broader knowledge source Distinctive microbial metabolomic knowledge Intelligent, query-capable Q&A Integrated microbial analysis	Limited included range of microbial species
	BV-BRC (Olson et al., 2023)		
	BacDive (Schober et al., 2025)		
	hGMB (Liu et al., 2021)		
	MicrobeWiki (Josowitz, n.d.)		
Microbe Knowledge Graph	KG4NH (Fu et al., 2023)	Standardized knowledge structure Broader knowledge source Scalable reasoning system Open-access web platform application	-
	FGMDI (Zhang et al., 2024)		
	MMiKG (Sun et al., 2023)		
	KetPath (Liu T. et al., 2024)		
	KGNMDA (Jiang et al., 2022)		
	KGVHI (Pan et al., 2024)		
Bioinformatics Platform	iMAP (Buza et al., 2019)	Synergistic interaction among microbial knowledge systems Zero coding web platform	Only have 16S rRNA sequencing analysis pipeline
	Majorbio Cloud (Ren et al., 2022)		
	Sangerbox (Shen et al., 2022)		

Despite its considerable strengths, IMicAP faces several critical limitations that must be addressed to fully realize its potential.

(1) The current version of IMicAP contains 594 microbial species (589 bacteria, 4 archaea, and 1 fungus). While we will continue to expand bacterial coverage in ongoing updates, the next version will prioritize substantial enrichment of archaea and fungi, with viral datasets to be included in a subsequent release. Furthermore, although the current version mainly integrates data at the species level, the next version plans to incorporate more detailed strain-level information to support higher-resolution analyses. To facilitate this progressive expansion, establishing a sustainable framework for long-term knowledge base maintenance is essential. Such a framework will enable the systematic integration of new research findings, coupled with rigorous quality control measures to ensure data accuracy. Given the existing mature data processing pipeline, the implementation of these future updates is not expected to pose major technical challenges.
(2) We fully acknowledge that knowledge extraction based on natural language models may inevitably suffer from model biases, which can lead to incomplete knowledge, weakly related associations, or even incorrect entries. This is a key reason why we introduced confidence scores, namely to allow users to assess the reliability of the extracted knowledge. Furthermore, the current knowledge framework of IMicAP is structured with microorganisms as its central focus. While this design enhances specialization within the microbial domain, it imposes certain limitations on the extensibility of knowledge representation. For example, direct associations between small molecules and diseases are currently absent (Markossian et al., 2018), with relations instead mediated through the intermediate path of disease-microbe-small molecule. In future updates, we aim to address these limitations by incorporating more comprehensive databases and conducting deeper literature mining.
(3) The Knowledge Graph module in IMicAP currently enables fundamental path-based reasoning. Although this method offers clear interpretability and reliable accuracy, it is constrained by high computational demands and restricted scalability. To overcome these limitations, future iterations of the platform will implement reasoning based on knowledge graph embeddings (Zhu et al., 2022). This advanced approach will combine confidence score and multi-omics information, including microbial gene expression profiles, GO categories, disease-related descriptions, protein sequences, and SMILES notations of small molecules, thereby substantially enhancing both the performance and scalability of the reasoning process (Zhu et al., 2023).
(4) The current version of IMicAP operates primarily as a knowledge-driven platform, with only the 16S rRNA Sequencing Analysis module involving real-world microbial data. Although we also plan to incorporate the Shotgun Metagenomic Sequencing (Quince et al., 2017) system in the next version, it still remains a limitation that carries a distinctive feature. In the era of big data, many microbial mechanisms exist in a “black-box” form that cannot be fully explained through established microbial knowledge alone, yet can be uncovered through large-scale microbial cohort data (Qin et al., 2025). Therefore, in future iterations, we plan to integrate existing databases to establish a dual-engine framework driven by both knowledge and data.

## 5 Conclusion

Collectively, we have developed IMicAP, a first comprehensive, open-access, AI-powered microbial platform designed to bridge the gap between fragmented microbial data and actionable biological insights. It directly addresses 4 key limitations in existing resources: fragmented analysis without integrated workflows, narrow disease scope, incomplete knowledge from restricted data sources, and the neglect of metabolomic information. By integrating 5 core modules within a unified environment, IMicAP facilitates hypothesis generation, multi-omics integration, and user-friendly data exploration. Looking ahead, continued expansion of microbial coverage, enhancement of reasoning scalability through embedding-based methods, and evolution toward a dual knowledge-and-data-driven architecture will further empower IMicAP to support mechanistic discovery in microbial systems biology and translational applications. As an open-access, web-based system, IMicAP stands as a versatile and evolving resource poised to accelerate research in microbial ecology, host–microbe interactions, and precision medicine.

## Data Availability

The datasets presented in this study can be found in online repositories. The names of the repository/repositories and accession number(s) can be found in the article/Supplementary material.
