# Supplementary material for: IMicAP: an intelligent microbial application platform for knowledge-driven microbiome research
Source: Front Microbiol. 2026 Oct 1;17:1955928. doi: 10.3389/fmicb.2026.1955928 (PMC13668525; doi:10.3389/fmicb.2026.1955928)
Supplement: Supplementary file 1 [file Table_8.docx]

Intelligent Microbial Application Platform (IMicAP) Operation Guidelines

# I. Guest / User Login

## 1) Guest Mode

(1) Access the platform URL; by default, you enter the homepage in guest mode (Figure 1).

In guest mode, the [Knowledge Graph] and [16S Sequencing Analysis] functions are not yet available; all other functions can be used normally.

Figure 1. IMicAP homepage (not logged in)

(2) Click the [Login] button, and the system will redirect to the login page (Figure 2).

Figure 2. Login page

## 2) User Login

(1) Enter the correct account and password, and enter the correct answer to the graphical CAPTCHA (Figure 3).

Figure 3. User login page

(2) Click [Login Now]; the page redirects to the platform homepage and displays the registered user's nickname, indicating a successful login (Figure 4).

Figure 4. IMicAP page after login

# II. Knowledge Retrieval and Viewing (Knowledge Query)

## 1) Knowledge Query (Figure 5)

Figure 5. Cation of the Knowledge Query function

The platform provides flexible and efficient intelligent query functions. Users can locate data through the following multiple methods:

A) **Exact match:** Enter a complete and accurate keyword, and the system returns data entries that exactly match it. Entering 'Escherichia coli' returns precise information about E. coli (Figure 6).

Figure 6. Exact query

**B) Tokenized search: Enter** a query containing multiple words; the system automatically splits it into independent keywords for a combined search, comprehensively covering related content. Entering 'coli', the system retrieves information containing 'coli' (Figure 7).

Figure 7. Tokenized query

**C) Substring match:** Even if you only remember part of a keyword, entering any continuous substring (at least 2 characters) allows the system to retrieve the complete keyword containing that fragment and related data. Entering 'richia' or 'escher', etc., retrieves results related to 'Escherichia coli' (Figure 8).

Figure 8. Substring query

**D) Fault-tolerant search**: When a keyword has minor spelling deviations, the system supports fuzzy matching with an edit distance of up to two characters (e.g., misspellings, missing characters, extra characters), effectively improving query fault tolerance. For example, entering 'Escherihia coli' (the order of 'r' and 'i' is wrong) or 'Eschrichia coli' (missing 'e'), the system can still correctly recognize and return results for 'Escherichia coli' (Figure 9).

Figure 9. Tolerant query

## 2) Categorized Query

**A) Gene Search**

The platform supports flexible retrieval through multiple dimensions of Gene identifiers. Users can enter fields such as gene's symbol, synonyms, NCBI_gene_id, dblinks, current_scientific_name, strains_tax_id, and substrains_tax_id in the search box. Selecting Gene in the search results displays gene-related information in the results (Figure 10).

Figure 10. Gene search results

B) **RNA Search**

Users can search via fields such as RNA's RNA_name, synonyms_of_RNA, locustag, gene_symbol, NCBI_gene_id, dblinks_gene, current_scientific_name, substrains_tax_id, and strains_tax_id in the search box. Selecting RNA in the search results displays only RNA-related information (Figure 11); clicking the target RNA opens its detail page.

Figure 11. RNA search

C) **Protein Search**

You can search via fields such as protein's uniprot_id, entry_name, gene_ordered_locus_names, gene_ORF_names, gene_names_(primary), gene_synonyms, enzyme_name, EC_number, dblinks_of_protein, dblinks_of_gene, protein_synonyms, current_scientific_name, substrains_tax_id, and strains_tax_id in the search box. Selecting protein in the search results displays only protein-related information (Figure 12); clicking opens the detail page for the target protein. Click the sidebar to view specific data for different parts of the protein (Figure 13).

Figure 12. Protein search

Figure 13. Protein categories in the left sidebar

D) **Disease Search**

You can search via fields such as disease's disease_name, synonym, and database_cross_reference in the search box. Selecting disease in the search results displays only disease-related information (Figure 14); clicking the target disease opens its detail page.

Figure 14. Disease search

**(5) Small molecule search**

You can search via fields such as small_molecule's Main Name, MCID, PMOID, Synonym, and Database_Cross_Reference in the search box. Selecting small molecule in the search results displays only small-molecule-related information (Figure 15); clicking the card opens the corresponding detail page.

Figure 15. Small molecule search

# III. Knowledge Graph Retrieval (Knowledge Graph)

## 1) The platform homepage provides a standalone 'Knowledge Graph' entry; clicking it enters the graph exploration interface (Figure 16)

Figure 16. Location of the Knowledge Graph function on the homepage

## 2) On the right of Adjacent, enter the search keyword and the entity type to be retrieved (Figure 17)

Figure 17. Adjacent search example

## 4) On the right of keyword-to-category, enter the [Keyword] and [Entity Type] (Figure 18)

Figure 18. Keyword-to-category search example

### 5) On the right of keyword-to-keyword, enter the [Keyword] and [Entity Type] (Figure 19)

Figure 19. keyword-to-keyword search example

# IV. Genome Browser

The platform provides two ways to access the genome browser: users can either access it directly through the general entry in the top navigation bar of the homepage (Figure 20), or, when querying a specific gene, enter directly from the 'Genome Browser' related entry within the corresponding detail page (Figure 21).

Figure 20. Location of the Genome Browser function on the homepage

Figure 21. Gene-end Genome Browser entry

Users can see the location of the target gene via the red indicator as follows (Figure 22):

Figure 22. Genome Browser display of gene ybjG

# V. 16S Analysis Sequencing Analysis (Figure 23)

Figure 23. Sequencing analysis entry on the homepage

Currently, the platform's 16S analysis cloud includes a total of 8 categories of analysis modules (sequence processing + standard analysis), with more than 30 analysis methods (Figure 24).

Figure 24. Overview of 16S sequencing analysis

# VI. Intelligent Q&A

This platform is equipped with a professional gut microbiota intelligent Q&A system that can accurately analyze users' complex scientific questions about microbial composition, microbiota functions, and host interactions, and provide authoritative answers based on literature and databases, helping users quickly acquire in-depth knowledge.

Users can click the [Intelligent Q&A] button on the website homepage to enter the system's main interface.

In the left sidebar of the Q&A page, users can conveniently view, create, delete, or switch historical sessions, enabling effective organization and management of conversation content (Figure 25).

Figure 25. Q&A page entry

# VII. Data Summary

## 1) Entity-Relationship Overview

### 1.1 Location and Layout (Figure 26)

**Location:** the top area of the page

**Layout**: divided into left and right sections, displaying 'Entities' and 'Relations' respectively

**Display form:** each section contains multiple statistical cards, with up to 6 cards per row

Figure 26. entity-relationship statistics

### 1.2 Entity Statistics (Entities)

**Function description:** displays the total statistics of various data entities on the platform

**Card content**:

Top: entity type name (e.g., Microbe, Gene, etc.)

Middle: total count of entities of this type (displayed in large font for emphasis)

### 1.3 Relationship Statistics (Relations)

**Function description: disp**lays the quantity statistics of relationships between different entities

**Card content:**

Top: relationship type name

Middle: total count of relationships of this type

## 2) Visualization Chart Area

### 2.1 Basic Operations

Layout: charts are arranged in a two-column grid, each column 50% wide

Container: each chart area contains a title bar and a content area, with a shadow effect to enhance visual hierarchy

### 2.2 Chart Type Description

The page contains three types of visualization charts, distinguished by different colors and styles:

### 2.2.1 Lollipop Chart (Figure 27)

**Identifie**r: circular endpoint + horizontal line

**Applicable data:** comparison of quantities of categorical data

**Interactive functi**ons:

Hovering the mouse over a dot displays the specific value

Clicking a dot shows detailed information (if a link is configured)

The left vertical axis text is clickable and has the same interactive effect

Figure 27. Lollipop chart

#### 2.2.2 Pie Chart (Figure 28)

**Identifier:** circular divided sectors

**Applicable data: pro**portion distribution data

**Interactive functions:**

Hovering over a sector displays the category's value and percentage

Clicking a sector or legend label triggers related actions

Equipped with leader lines and external labels to avoid label overlap

Figure 28. Pie chart

#### 2.2.3 Histogram (Figure 29)

**Identif**ier: bars + top dots

**Applicable data:** distribution of continuous data

**Interactive functions:**

Hovering over a bar displays the value range and frequency

Clicking a bar or dot enables in-depth exploration

Automatically handles boundary value display (e.g., '>X' or '<Y')

Figure 29. Histogram

### 2.3 Chart Category Details (Figure 30, Figure 31)

**Category 1: Taxonomy Statistics**

**Includes** 8 levels: Subkingdom, Kingdom, Phylum, Class, Order, Family, Genus, Species

The first two levels use pie charts; the last six levels use lollipop charts

Figure 30. Taxonomy statistics - pie chart

Figure 31. Taxonomy statistics - lollipop chart

**Category 2: Protein Statistics (Figure 32)**

**Single tab:** Protein type distribution

Figure 32. Protein statistics

**Category 3: RNA Statistics (Figure 33)**

Single tab: RNA type distribution

Figure 33. RNA statistics

**Category 4: Morphology Features (Figure 34)**

6 subcategories: flagellar arrangement, Gram staining, motility, cell shape, cell width, cell length

Figure 34. Morphology features

**Category 5: Cultivation Conditions (Figure 35)**

9 subcategories: medium, pH growth range, optimal pH, temperature growth range, temperature range, optimal temperature, oxygen tolerance, salt growth range, optimal salinity

Figure 35. Cultivation conditions

**Category 6: Metabolic Features (Figure 36)**

7 subcategories: metabolite utilization, metabolite production, enzyme activity, antibiotic susceptibility, antibiotic resistance

Figure 36. Metabolic features

**Category 7: Isolation Sources (Figure 37)**

Single tab: country distribution

Figure 37. Isolation sources

**Category 8: Molecular Biology & Pathogenicity (Figure 38)**

3 subcategories: GC content, pathogenicity, risk level

Figure 38. Molecular biology & pathogenicity

**Category 9: Tests & Profiles (Figure 39)**

4 subcategories: fatty acid profile, Voges-Proskauer test, methyl red test, indole test

Figure 39. Tests & profiles
