# Supplementary material for: IMicAP: an intelligent microbial application platform for knowledge-driven microbiome research
Source: Front Microbiol. 2026 Oct 1;17:1955928. doi: 10.3389/fmicb.2026.1955928 (PMC13668525; doi:10.3389/fmicb.2026.1955928)
Supplement: Supplementary file 2 [file Table_9.docx]

**Comparison of Intelligent QA**

A. **IMicAP (Qwen3-Max-Thinking-Preview + IMicAP RAG)**

- **System Prompt:** You are a gut microbiome expert. Please answer the question based on the context retrieved from your knowledge base.
- **Embedding Model:** Qwen3-Embedding-8B
- **Context Window:** 262K tokens
- **Temperature:** 0.7
- **Chunking Strategy:** Chunking is done at the entity/relation level: each microbe and its associated relations (taxonomy, metabolism, clinical aspects, diseases, small molecules, with confidence scores and PMIDs) is an atomic retrieval unit, returning complete entries instead of arbitrary text windows.
- **Retrieval Depth:** 5

B. **Qwen3-Max-Thinking-Preview Only**

- **System Prompt:** None.
- **Embedding Model:** Qwen3-Embedding-8B
- **Context Window:** 262K tokens
- **Temperature:** 0.7

**Question 1: Introduce about Escherichia coli.**

**Answer of A:**

✅ **Introduction to Escherichia coli (E. coli)**

Escherichia coli (commonly abbreviated as E. coli) is a Gram-negative, rod-shaped, facultative anaerobic bacterium that is commonly found in the lower intestine of warm-blooded organisms, including humans. Most strains are harmless and even beneficial, forming part of the normal gut microbiota by producing vitamin K₂ and preventing colonization by pathogenic bacteria.

**Taxonomy**

Domain: Bacteria

Phylum: Pseudomonadota (formerly Proteobacteria)

Class: Gammaproteobacteria

Order: Enterobacterales

Family: Enterobacteriaceae

Genus: Escherichia

Species: E. coli

**Characteristics**

Morphology: Straight rods, 1–3 µm long, with peritrichous flagella (motile).

Metabolism: Facultative anaerobe — can grow with or without oxygen.

Growth: Optimal at 37°C (human body temperature), grows well on standard lab media like MacConkey agar or LB broth.

Habitat: Primarily colonizes the gastrointestinal tract shortly after birth; also found in environment (soil, water) via fecal contamination.

**Pathogenic Strains**

While most E. coli are commensals, some strains are pathogenic and cause disease:

| **Pathotype** | **Disease** | **Key Features** |
| --- | --- | --- |
| ETEC (Enterotoxigenic) | Traveler’s diarrhea | Produces heat-labile (LT) and/or heat-stable (ST) enterotoxins |
| EPEC (Enteropathogenic) | Infantile diarrhea | Attaches to intestinal cells, causes “attaching and effacing” lesions |
| EHEC/STEC (Shiga-toxin producing) | Hemorrhagic colitis, HUS | Produces Shiga toxin (e.g., O157:H7 strain); undercooked beef, raw milk |
| EIEC (Enteroinvasive) | Bacillary dysentery-like | Invades colonic epithelium, mimics Shigella |
| EAEC (Enteroaggregative) | Persistent diarrhea | “Stacked-brick” adherence pattern; common in children in developing countries |
| UPEC (Uropathogenic) | Urinary tract infections | Adhesins (P fimbriae), hemolysin, biofilm formation |

**Role in Biotechnology & Research**

One of the most widely studied model organisms in molecular biology.

Used for recombinant protein production (e.g., insulin, growth hormone).

Tool for genetic engineering, synthetic biology, and metabolic engineering.

Laboratory workhorse due to fast growth, well-characterized genetics, and ease of manipulation.

**Ecology & Public Health**

Indicator organism for fecal contamination in water and food.

Antibiotic resistance in E. coli (e.g., ESBL, carbapenemase producers) is a major global health threat.

Surveillance and control of multidrug-resistant (MDR) E. coli is critical in hospitals and agriculture.

**Note Regarding Provided Context**

The text you shared discusses Campylobacter coli, which:

Is curved/S-shaped, microaerophilic, requires special culture conditions.

Causes campylobacteriosis (gastroenteritis), often from poultry.

Belongs to Epsilonproteobacteria — evolutionarily distant from E. coli (Gammaproteobacteria).

They should not be confused — despite both being Gram-negative gut-associated pathogens, they differ significantly in biology, clinical impact, and treatment.

**Summary**

Escherichia coli is a versatile, well-studied bacterium — mostly harmless, sometimes deadly. It plays vital roles in human health, industry, and science. Pathogenic variants remain a major public health concern worldwide, especially with rising antimicrobial resistance.

Let me know if you’d like details on specific strains, virulence factors, or its role in IBD or probiotics!

**Answer of B:**

**Escherichia coli (E. coli)**

Escherichia coli, commonly known as E. coli, is a Gram-negative, rod-shaped bacterium that is commonly found in the lower intestine of warm-blooded organisms. It was first identified by Theodor Escherich in 1885 and named after him.

**Characteristics**

- Shape and structure: Rod-shaped (bacillus), facultative anaerobic bacterium with flagella for movement
- Size: Typically 1-3 micrometers in length
- Growth: Can reproduce rapidly under favorable conditions, with a generation time of about 20 minutes

**Natural Habitat**

Most E. coli strains are harmless and are actually an important part of a healthy human intestinal tract, where they:

- Aid in digestion
- Produce vitamin K2
- Prevent colonization by pathogenic bacteria

**Pathogenic Strains**

While most strains are harmless, some have acquired virulence factors that make them pathogenic:

- Enterohemorrhagic E. coli (EHEC): Strain O157:H7 can cause severe food poisoning with symptoms including bloody diarrhea and potentially hemolytic uremic syndrome (HUS)
- Enteropathogenic E. coli (EPEC): Causes infant diarrhea in developing countries
- Enterotoxigenic E. coli (ETEC): Common cause of traveler's diarrhea
- Uropathogenic E. coli (UPEC): Major cause of urinary tract infections

**Scientific Importance**

E. coli is one of the most extensively studied prokaryotic model organisms and has been instrumental in:

- Molecular biology research
- Genetic engineering
- Biotechnology applications
- Understanding fundamental biological processes

**Prevention and Control**

Pathogenic E. coli infections are typically prevented through:

- Proper food handling and cooking
- Clean water supplies
- Good personal hygiene
- Appropriate sewage treatment

Antibiotic treatment is used for serious infections, though antibiotic resistance is becoming an increasing concern with some E. coli strains.

E. coli remains both a vital component of our gut microbiome and, in certain pathogenic forms, a significant public health concern worldwide.

**Question 2: Which microorganisms are more likely to cause infection?**

**Answer of A:**

Microorganisms that are more likely to cause infection include opportunistic and obligate pathogens that can exploit disruptions in host defenses, microbial balance, or anatomical barriers. Based on the context provided, Parvimonas micra (P. micra) is one such microorganism with significant pathogenic potential.

**Key Characteristics of Parvimonas micra That Increase Its Likelihood to Cause Infection:**

**Commensal Pathogen:**
P. micra normally resides as part of the human microbiota — primarily in the subgingival dental plaque of the oral cavity and also in the gastrointestinal tract. Under normal conditions, it exists in balance with other microbes. However, when this balance is disturbed (e.g., due to poor oral hygiene, immune suppression, or tissue damage), its abundance can increase, leading to disease.

**Biofilm Association:**
Although P. micra cannot initiate biofilm formation, it colonizes pre-established biofilms — particularly in the oral cavity. Biofilms protect bacteria from host immune responses and antibiotics, enhancing their persistence and virulence.

**Obligate Anaerobe:**
Its anaerobic nature allows it to thrive in low-oxygen environments like deep periodontal pockets or abscesses, where it can evade immune surveillance and proliferate.

**Polymicrobial Synergy:**
P. micra often acts in concert with other pathogens (e.g., Aggregatibacter aphrophilus) to cause severe infections such as pleural empyema, indicating its role in synergistic, polymicrobial infections.

**Systemic Translocation Potential:**
It can translocate from its native niches (oral cavity or GI tract) to distant sites via bloodstream or direct spread, causing:

Endocarditis

Septic arthritis

Intracranial abscesses

Peritonsillar abscesses

Spondylodiscitis

Pericarditis

Septic embolism

**Virulence via Ecological Disruption:**
Even slight increases in its abundance within biofilms can disrupt microbial homeostasis, triggering inflammation and tissue destruction — hallmark features of periodontitis.

**General Microbial Traits That Increase Infection Risk (Beyond P. micra):**

**While the context focuses on P. micra, broadly speaking, microorganisms more likely to cause infection typically exhibit:**

Adhesion factors (e.g., pili, adhesins) for attachment to host tissues

Invasion mechanisms to penetrate epithelial barriers

Toxin production (exotoxins/endotoxins) that damage host cells

Immune evasion strategies (capsules, antigenic variation)

Antibiotic resistance genes

Ability to form biofilms

Facultative or obligate intracellular survival

**Examples beyond P. micra include:**

Staphylococcus aureus (skin/soft tissue, systemic infections)

Escherichia coli (UTIs, sepsis)

Pseudomonas aeruginosa (hospital-acquired pneumonia, burn wound infections)

Candida albicans (fungal opportunistic infections)

**Conclusion:**

Among the microorganisms described, Parvimonas micra stands out as a clinically relevant pathogen with increasing recognition for causing both localized (periodontitis) and systemic (endocarditis, septic arthritis) infections. Its ability to thrive in biofilms, act synergistically with other pathogens, and translocate systemically makes it particularly dangerous under permissive host conditions. Therefore, microorganisms like P. micra — commensals with pathogenic potential under dysbiotic or immunocompromised conditions — are more likely to cause infection when ecological or immune barriers are compromised.

**Answer of B:**

**The microorganisms most likely to cause infections are:**

**Bacteria (e.g., E. coli, Staph)**

Why: They are the most common cause of everyday infections.

Examples: Strep throat, urinary tract infections (UTIs), skin abscesses, and food poisoning.

Treatment: Usually cured with antibiotics.

**Viruses (e.g., Flu, Cold, COVID-19)**

Why: They spread very easily through the air or contact and mutate quickly.

Examples: Colds, flu, fever, and coughs.

Treatment: Antibiotics do not work; prevention relies on vaccines and your own immune system.

**Two other types:**

Fungi (e.g., Athlete's foot, Yeast infections): Mostly infect people with weakened immune systems.

Parasites: Usually caught from contaminated water or food.

Bottom Line: In daily life, bacteria and viruses are the main culprits. However, if your immune system is weak, fungi can also become a serious threat.
